# Case Report: Refractory life-threatening post-biopsy hemorrhage in a neonate successfully controlled with Ankaferd Blood Stopper

**DOI:** 10.3389/fped.2026.1846312

**Published:** 2026-09-16

**Authors:** Timucin Imdadoglu, Bulent Guzel, Senay Coskun, Asli Okbay Gunes, Emel Altuncu

**Affiliations:** 1 Department of Pediatrics, Sancaktepe Prof. Dr. Ilhan Varank Training and Research Hospital, Istanbul, Turkiye; 2 Division of Neonatology, Department of Pediatrics, Sancaktepe Prof. Dr. Ilhan Varank Training and Research Hospital, Istanbul, Turkiye; 3 Division of Neonatology, Department of Pediatrics, Sancaktepe Prof. Dr. Ilhan Varank Training and Research Hospital, Istanbul, Turkiye; 4 Department of Neonatology, University of Health Sciences, Sancaktepe Prof. Dr. Ilhan Varank Training and Research Hospital, Istanbul, Turkiye; 5 Division of Neonatology, Department of Pediatrics, Sancaktepe Prof. Dr. Ilhan Varank Training and Research Hospital, Istanbul, Turkiye

**Keywords:** Ankaferd Blood Stopper, biopsy-related bleeding, infantile undifferentiated round-cell sarcoma, life-threatening hemorrhage, newborn

## Abstract

**Introduction:**

Biopsy-related hemorrhage in neonates is rare but may rapidly become life-threatening when refractory to conventional hemostatic interventions. This case is unique in reporting the first successful use of topical Ankaferd Blood Stopper® (ABS) as a salvage hemostatic agent in a neonate with infantile undifferentiated round-cell sarcoma within the differential spectrum of BCOR-altered sarcomas, an exceptionally rare malignancy, following life-threatening, refractory post-biopsy hemorrhage.

**Patient concerns and clinical findings:**

A term female neonate presented with a rapidly enlarging left facial mass with proptosis and restricted eye movements. Intraoral incisional biopsy was performed to obtain histopathological diagnosis. Within 72–74 h post-biopsy, active hemorrhage persisted despite multiple conventional hemostatic measures, including local compression, topical epinephrine, intravenous tranexamic acid, vitamin K, and fresh frozen plasma (FFP). The neonate developed hemorrhagic shock with hemodynamic instability (heart rate >170 bpm, blood pressure 45/25 mmHg, oxygen saturation <88%) and required packed red blood cell (PRBC) transfusion. Baseline coagulation parameters were within normal neonatal reference ranges; comprehensive coagulopathy screening excluded an underlying bleeding disorder.

**Diagnosis, intervention, and outcomes:**

Histopathological examination was most consistent with infantile undifferentiated round-cell sarcoma within the differential spectrum of BCOR-altered sarcomas. Topical ABS was applied as a salvage intervention at 72–74 h post-biopsy. Hemostasis was achieved within minutes and was reproducible across three applications, with a total administered volume of no more than 6 mL and no local or systemic adverse effects. The neonate subsequently underwent urgent surgical debulking/resection and adjuvant therapy. At six-month follow-up, the patient remains alive without evidence of disease recurrence.

**Conclusion:**

Topical ABS may represent an effective and potentially lifesaving option for managing severe, refractory hemorrhage in neonates when conventional therapies fail. Its coagulation-independent mechanism may be particularly advantageous in this vulnerable population. This case highlights the importance of considering ABS in life-threatening neonatal hemorrhagic emergencies and underscores the need for prospective studies to establish standardized neonatal protocols.

## Introduction

Post-biopsy bleeding is a rare complication in neonates without underlying hemostatic disorders and is usually manageable with local compression and topical agents. However, when driven by an aggressive, highly vascular underlying pathology, bleeding can rapidly become massive and life-threatening. Neonates are particularly vulnerable due to immature coagulation pathways, physiologically lower levels of vitamin K-dependent coagulation factors, and limited physiological reserves (1, 2).

Ankaferd Blood Stopper® (ABS) is a standardized herbal extract approved by the Turkish Ministry of Health as a topical hemostatic agent. Its mechanism involves the rapid formation of a protein network that facilitates erythrocyte aggregation and clot formation, independent of the classical coagulation cascade. This coagulation-independent mechanism may be particularly advantageous in clinical situations where conventional hemostatic measures are insufficient (3, 4).

Infantile undifferentiated round-cell sarcoma within the differential spectrum of BCOR-altered sarcomas represents an extremely rare soft tissue malignancy predominantly affecting infants and young children; neonatal presentation is exceptionally uncommon (5–7). Its highly vascular nature poses significant procedural risks, and life-threatening hemorrhagic complications following biopsy have not previously been described in this entity.

Herein, we report the first documented use of topical ABS as a salvage hemostatic intervention in a term neonate with refractory, life-threatening post-biopsy hemorrhage attributable to an infantile undifferentiated round-cell sarcoma within the differential spectrum of BCOR-altered sarcomas, unresponsive to all conventional measures.

## Case description

### Patient information

A term female neonate was born at 37 weeks’ gestation, weighing 3,265 grams, via cesarean section to a 24-year-old primigravida with preeclampsia. The mother had intermittent antenatal follow-up, predominantly conducted at another institution; structured fetal anomaly scan records were not available for review. No fetal facial or orbital abnormality was documented in the available antenatal records. The neonate was born in good condition and discharged at 48 h of life without any dysmorphic features or palpable mass. The family first noticed a left cheek swelling, described as approximately chickpea-sized, at postnatal day 5 (115th postnatal hour), prompting emergency department presentation. The neonate was admitted to the neonatal intensive care unit (NICU) for hyperbilirubinemia requiring phototherapy. No prior interventions for the facial mass had been performed.

Family history was notable for hemangioma in one maternal relative; a pulmonary cyst requiring surgical resection and thyroid nodular disease in another maternal relative; autosomal dominant polycystic kidney disease requiring nephrectomy in the father; and thyroid nodular disease requiring thyroidectomy in the paternal grandmother. No consanguinity was reported. Germline testing for tumor predisposition syndromes was not performed.

### Clinical findings

On admission at postnatal day 5, vital signs were: heart rate 142 bpm, respiratory rate 44/min, temperature 36.1 °C; weight 3,265 g (AGA), length 50 cm, head circumference 36 cm. The anterior fontanel was open and normotensive (1 × 1 cm). No cleft lip or palate was present; bilateral choanae were patent. Cardiac auscultation revealed normal heart sounds without an audible murmur; echocardiography demonstrated a small muscular ventricular septal defect (left-to-right shunt; Pmax 40 mmHg) and a patent foramen ovale, both hemodynamically insignificant. Abdominal examination revealed no hepatosplenomegaly. Neurological examination was age-appropriate. Ophthalmologic consultation at admission documented normal bilateral fundoscopic findings with equal and normal-caliber retinal vasculature; restriction of left supraduction and adduction with progressive proptosis developed on subsequent examinations as the mass enlarged.

At initial presentation, color Doppler ultrasonography demonstrated a solid lesion adjacent to the posterior aspect of the left parotid gland, extending into the deep tissue planes, with internal color-flow signals and small cystic spaces. A vascular lesion, including infantile hemangioma, was initially considered. The lesion was monitored with repeated bedside ultrasonography during the NICU stay, and progressive enlargement was noted, although separate formal reports for these serial examinations were unavailable. Two contrast-enhanced MRI examinations were subsequently obtained. The first MRI, performed on postnatal day 6, demonstrated a 35 × 36 mm intensely enhancing lesion arising from the left pterygoid muscles and extending into the orbital and temporal fossae (Figure 1). A second MRI obtained approximately 10–11 days later showed enlargement to approximately 39 × 38 mm, with increasing mass effect, optic nerve displacement, and extension into the adjacent intraorbital and intracranial compartments. Clinically, the neonate developed progressive proptosis, restricted eye movements, and signs of visual compromise.

**Figure 1:**
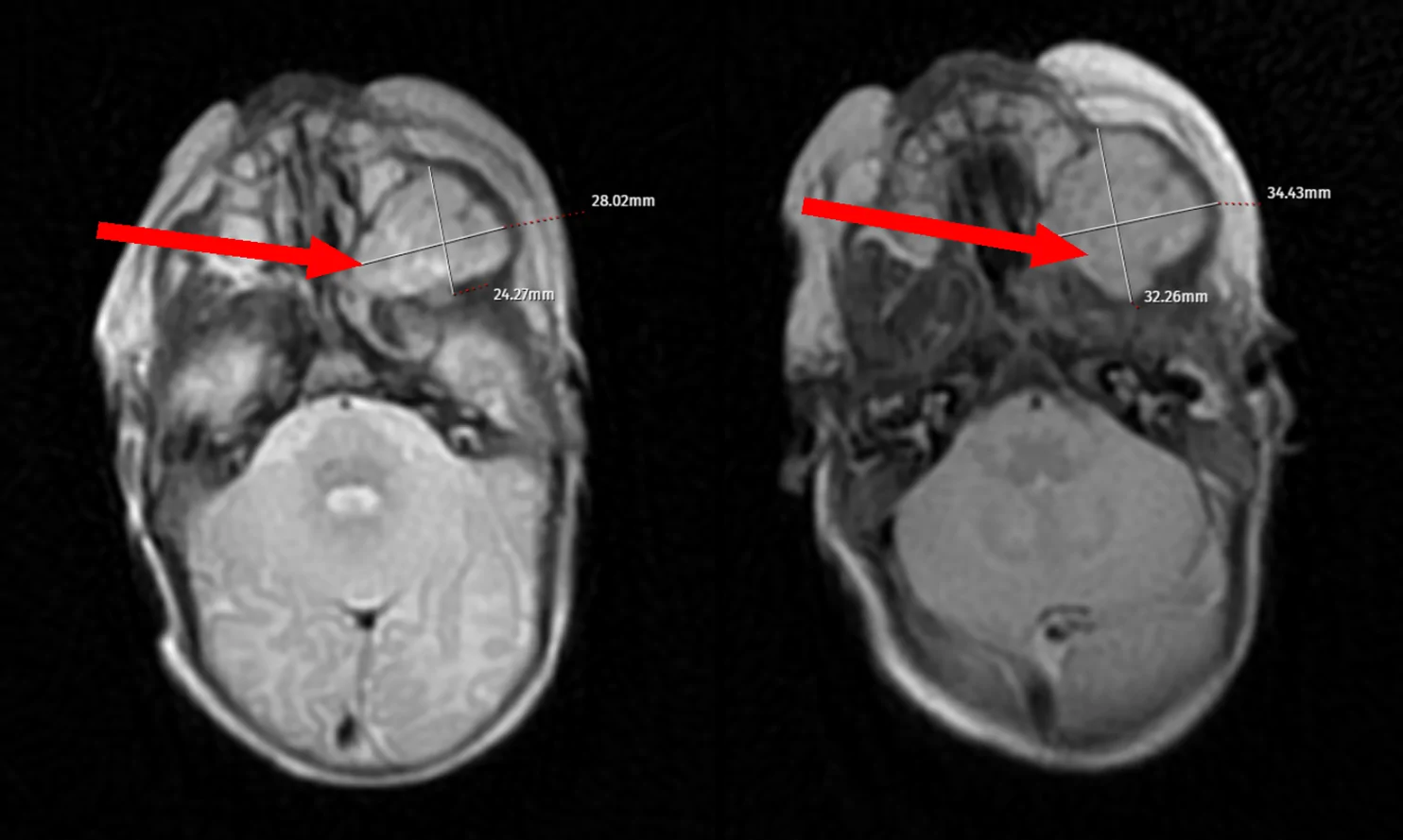
Contrast-enhanced magnetic resonance imaging of the lesion. **(A)** Axial view demonstrating an intensely enhancing mass originating from the left pterygoid region with extension toward the orbit. **(B)** Axial view showing orbital involvement and displacement of adjacent structures. Arrows indicate the tumor.

The lesion was initially suspected to be infantile hemangioma; however, rapid growth, atypical features (firm consistency, deep extension), and progressive visual compromise raised concern for malignancy. Differential diagnoses included infantile hemangioma, rhabdomyosarcoma, neuroblastoma, and other soft tissue sarcomas. Hemangioma screening at other body sites was negative. Given progressive clinical deterioration and the need for histopathological confirmation, biopsy was deemed necessary.

### Timeline

Table 1.

**Table 1:** Timeline of Key clinical events.

Postnatal day/time	Event	Key findings
Day 0	Birth	Term, 3,265 g, cesarean section
Day 5	NICU admission	Hyperbilirubinemia; left cheek swelling
Day 6	Initial MRI	35 × 36 mm enhancing lesion, left pterygoid region
Approximately 10–11 days later	Second contrast-enhanced MRI	Enlargement to 39 × 38 mm; increasing mass effect, optic nerve displacement, intraorbital and intracranial extension
Day 20	Intraoral incisional biopsy	5 × 4 × 3 mm specimen; internal vascular flow on Doppler ultrasonography and intense enhancement on MRI
Hours 0–24 post-biopsy	Initial hemostasis	Minimal immediate bleeding; intermittent oozing begins
Hours 24–72 post-biopsy	Progressive hemorrhage	Bleeding increases; conventional measures fail
Post-biopsy hour 72–74	Hemorrhagic shock	Hb 7.5 g/dL; HR >170 bpm; BP 45/25 mmHg; SpO₂ < 88%; PRBC transfusion
Post-biopsy hour 72–74	1st ABS application	Hemostasis within minutes
Post-biopsy hour 86	Recurrent bleeding; 2nd ABS application	Hemostasis achieved
Post-biopsy hour 110	Recurrent bleeding; 3rd ABS application	Sustained hemostasis
Day 26	Histopathological diagnosis	Histopathological findings most consistent with infantile undifferentiated round-cell sarcoma within the differential spectrum of BCOR-altered sarcomas
Day 42 (Week 6)	Urgent surgical debulking/resection	Intraoperative bleeding controlled conventionally
Months 1–7	Adjuvant therapy	Chemotherapy (EpSSG RMS 2005); focal radiotherapy
6-month follow-up	Disease-free status	No recurrence; no further hemorrhage

ABS, Ankaferd Blood Stopper®; BP, blood pressure; Hb, hemoglobin; HR, heart rate; MRI, magnetic resonance imaging; NICU, neonatal intensive care unit; PRBC, packed red blood cell; SpO₂, peripheral oxygen saturation.

## Diagnostic assessment, therapeutic intervention, follow-up and outcomes

### Diagnostic assessment

Pre-biopsy laboratory investigations, including complete blood count and coagulation parameters, were within normal neonatal reference ranges (Table 2). Comprehensive coagulopathy screening including factor VIII, factor IX, von Willebrand factor activity, and D-dimer excluded an underlying inherited or acquired bleeding disorder (Table 2). Coagulation parameters remained within normal ranges during the hemorrhagic episode. The hemorrhage was attributed to the highly vascular nature of the underlying tumor.

**Table 2:** Laboratory parameters during clinical course and coagulopathy screening.

Parameter	Pre-biopsy (baseline)	During hemorrhage	Post-ABS	Reference range (neonate)
Hemoglobin (g/dL)	13.5–14.0	7.5	10–11	10.0–18.0
Hematocrit (%)	40–42	22–25	30–33	31.0–55.0
Platelet (×10^3^/*μ*L)	320	285	310	150–450
PT (sec)	14.6	14.2	14.8	13–18
INR	1.25	1.22	1.27	0.8–1.5
aPTT (sec)	38.9	37.5	39.1	30–60
Fibrinogen (mg/dL)	280	260	290	200–400
Factor VIII (%)	85	—	—	50–150
Factor IX (%)	72	—	—	50–150
von Willebrand factor activity (%)	92	—	—	50–150
D-dimer (μg/mL)	0.4	—	—	<0.5
Heart rate (bpm)	140–150	>170	140–150	120–160
Blood pressure (mmHg)	Normal	45/25 (hypotensive)	Stabilized	60–90/30–60
Oxygen saturation (%)	>95	<88	>95	>95

PT, prothrombin time; INR, international normalized ratio; aPTT, activated partial thromboplastin time. Coagulation factor levels and von Willebrand factor activity were measured pre-biopsy only. Dashes indicate not repeated at that time point.

Although internal vascularity and intense contrast enhancement were demonstrated, specific feeding vessels were not formally mapped or documented, and no dedicated angiographic or interventional radiology assessment was performed before biopsy.

Because of the rapid interval enlargement and increasing orbital and dural involvement, histopathological confirmation was considered necessary. Complete excision was not considered feasible because of the extensive anatomical involvement of the lesion.

### Therapeutic intervention

An open intraoral incisional biopsy of the clinically accessible buccal component was performed by the pediatric otorhinolaryngology team in the operating room under general anesthesia with endotracheal intubation (Figure 2).

**Figure 2:**
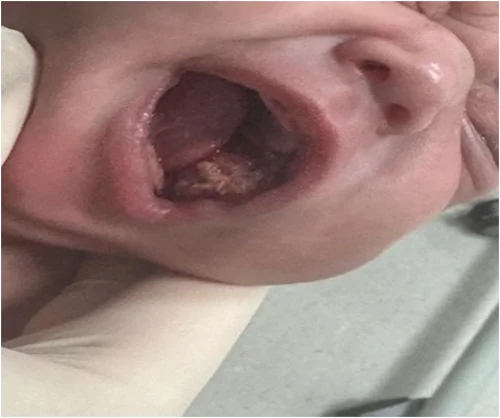
Intraoral view of the biopsy site in the left buccal region of the neonate. The image demonstrates the anatomically challenging location and limited accessibility of the bleeding focus. Arrow indicates the biopsy site. (Image published with parental consent; patient identity anonymized.).

Before the procedure, routine pre-anesthetic blood bank preparation was completed, including blood grouping, cross-matching, and preparation of packed red blood cells. Fresh frozen plasma was not pre-thawed but was available through the blood bank if clinically required. Intraoperative bleeding was minimal and was controlled with local measures. No clinically significant hemorrhage was observed during the procedure or immediately after the neonate returned to the NICU. The infant remained intubated after the procedure and was successfully extubated on the following day. Slow oozing from the biopsy site subsequently developed and progressively increased over the following hours. The pediatric otorhinolaryngology team reassessed the patient as bleeding worsened.

At 72–74 h post-biopsy, active hemorrhage persisted despite all conventional hemostatic measures: local compression, topical epinephrine, intravenous tranexamic acid (15 mg/kg), vitamin K (1 mg/kg intravenous), and FFP (10 mL/kg). Estimated cumulative blood loss was approximately 80–100 mL. The neonate developed hemorrhagic shock and required PRBC transfusion (15 mL/kg).

Following parental informed consent, topical ABS was applied as a salvage intervention at 72–74 h post-biopsy. The undiluted solution was used directly from the prefilled 10 mL syringe. A sterile gauze soaked with approximately 1–2 mL of solution was applied to the bleeding site with gentle compression for 2–3 min. Hemostasis was achieved within minutes of the first application.

Due to recurrent bleeding at 12 h and 36 h after the initial application, two additional applications were performed using the identical technique. Total administered volume across all three applications did not exceed 6 mL. The neonate was closely monitored for local complications (tissue necrosis, infection, mucosal discoloration) and systemic adverse effects (changes in vital signs and coagulation parameters). No adverse effects were observed following any of the three applications. Sustained hemostasis was achieved following the third application.

### Follow-up and outcomes

Initial abdominal ultrasonography showed no focal solid-organ lesion, intra-abdominal mass, or pathological lymphadenopathy, and transfontanelle ultrasonography was unremarkable. Following histopathological diagnosis, systemic staging was performed using thoracic MRI and contrast-enhanced upper and lower abdominal MRI. No pulmonary or mediastinal lesion, abdominal or pelvic mass, pathological lymphadenopathy, or focal lesion involving the liver, spleen, pancreas, kidneys, or adrenal glands was detected. The available systemic staging therefore showed no evidence of distant thoracoabdominal disease.

The neonate was referred to a pediatric oncology center following histopathological confirmation of infantile undifferentiated round-cell sarcoma within the differential spectrum of BCOR-altered sarcomas. At 6 weeks of age, urgent surgical debulking/resection was performed; intraoperative bleeding was moderate and controlled with conventional techniques without the need for ABS. The patient subsequently received chemotherapy according to the EpSSG RMS 2005 protocol and focal radiotherapy. At six months, no tumor recurrence or further hemorrhagic episode had been documented. No delayed adverse effects attributable to ABS were identified.

Histopathological examination of the resection specimen demonstrated a malignant mesenchymal tumor composed of myxoid spindle-cell areas and a primitive small round-cell component, with focal chondroid heterologous differentiation; immunohistochemistry showed a consistent immunophenotype including weak BCOR expression. Based on the morphological and immunohistochemical findings and external expert pathological consultation, the tumor was considered most consistent with infantile undifferentiated round-cell sarcoma with areas of infantile primitive mesenchymal tumor, within the differential spectrum of BCOR-altered sarcomas. An RNA-based sarcoma fusion panel did not identify a diagnostically relevant fusion or BCOR alteration; however, the negative result was considered nonconfirmatory because of low RNA read quality. Screening for congenital infections using a TORCH panel yielded negative results. Whole-exome sequencing, copy-number variation analysis, and constitutional germline testing were not performed.

## Discussion

Neonatal hemorrhage presents unique challenges due to the developmental immaturity of the hemostatic system. Although laboratory parameters may appear within normal ranges, the hemostatic response may be insufficient when confronted with a highly vascular lesion (1, 2). In the present case, all conventional hemostatic measures failed, and the neonate progressed to hemorrhagic shock, underscoring the severity of this clinical scenario and the limitations of standard interventions in this population.

Infantile undifferentiated round-cell sarcoma within the differential spectrum of BCOR-altered sarcomas is an extremely rare soft tissue malignancy predominantly affecting infants and young children; neonatal presentation is exceptionally rare (5–7). Its high vascularity likely accounts for the severity of hemorrhage observed in this case. This diagnosis should be considered in the differential of rapidly enlarging facial masses in neonates, particularly when conventional hemostatic measures fail following biopsy.

ABS promotes rapid formation of a protein network facilitating erythrocyte aggregation and clot formation, independent of the classical coagulation cascade. This mechanism may be particularly advantageous in neonates, in whom the immaturity of coagulation pathways may limit the efficacy of cascade-dependent agents. ABS has demonstrated hemostatic efficacy in various clinical settings, including pediatric tonsillectomy and adenoidectomy (8, 9), dental procedures (10, 11), cardiothoracic surgery (12), gastrointestinal bleeding (13, 14), and traumatic hemorrhage (15). Prior neonatal reports include infantile hemangioma-related bleeding (16), persistent nasal bleeding in premature neonates on continuous positive airway pressure (CPAP) (17), and acute pulmonary hemorrhage (18). Additional pediatric procedural literature includes skin biopsy techniques and associated considerations in the pediatric age group (19), as well as the use of ABS as a hemostatic agent in adenoidectomy and primary–tooth pulpotomy procedures (20–22).

### Strengths and limitations

The primary strength of this report is the detailed documentation of a reproducible hemostatic response across three ABS applications in a critically ill neonate, with no observed adverse effects, providing clinically relevant safety and efficacy data in a population for whom such data are scarce. The case also highlights a novel application context: tumor-related, life-threatening hemorrhage in the neonatal period.

Several limitations of this case report warrant acknowledgment. First, although color Doppler ultrasonography demonstrated intralesional flow, dedicated mapping of the feeding and draining vessels was not performed before biopsy, and pre-procedural embolization was not undertaken. Second, systemic staging was completed after histopathological diagnosis rather than at the initial presentation; subsequent thoracic and abdominal MRI showed no evidence of distant thoracoabdominal disease. Third, the consistent temporal response observed across three ABS applications supports an association with hemostasis; however, the absence of a controlled comparison precludes definitive causal attribution. Recurrent bleeding may have reflected continued oozing from the disrupted, highly vascular biopsy bed. Fourth, detailed antenatal imaging records were unavailable, preventing a definitive explanation for the prenatal non-detection of the lesion. Fifth, a formal multidisciplinary comparison of the open intraoral approach with image-guided percutaneous alternatives was not documented. In retrospect, dedicated vascular mapping and consideration of a limited image-guided biopsy might have reduced tissue disruption, although a substantial hemorrhage risk would still have remained.

### Take-away lessons

Topical ABS should be considered as a salvage hemostatic option in neonates with life-threatening hemorrhage refractory to conventional measures. Its coagulation-independent mechanism makes it particularly relevant when the classical cascade is insufficient. Further prospective studies and case series are needed to establish standardized protocols for neonatal use.

## Patient perspective

As the parents of the patient, we experienced an extraordinarily stressful period when our daughter developed severe, uncontrolled bleeding following the biopsy procedure. We were informed about the experimental nature of the Ankaferd Blood Stopper treatment and gave our written consent, as all other options had been exhausted. The rapid cessation of bleeding after the application brought us immense relief. We are grateful to the medical team for their prompt and decisive action. Our daughter has since undergone surgery and chemotherapy and is currently doing well. We hope that sharing this experience will help other families and clinicians in similar situations.

## Data Availability

The original contributions presented in the study are included in the article/Supplementary Material, further inquiries can be directed to the corresponding author.
